# Stacked hybrid model for load forecasting: integrating transformers, ANN, and fuzzy logic

**DOI:** 10.1038/s41598-025-04210-1

**Published:** 2025-06-04

**Authors:** Elakkiya E, Antony Raj S, Arunkumar Balakrishnan, Bhavyasri Sanisetty, Revanth Balaji Bandaru

**Affiliations:** 1 Department of Computer Science and Engineering, SRM University AP, Amaravati, Andhra Pradesh 522502 India; 2 Department of Electrical and Electronics Engineering, St. Anns College of Engineering and Technology, Chirala, Andhra Pradesh 523187 India; 3 Department of Information Technology, Manipal Institute of Technology Bengaluru, Manipal Academy of Higher Education, Manipal, India

**Keywords:** Load forecasting, Time series transformer, FLT + ANN, Hybrid model, Energy management, Electrical and electronic engineering, Energy grids and networks, Power distribution

## Abstract

Modern energy management systems must include load forecasting in order for utilities to plan and optimize electricity distribution, lower operating costs, and improve grid stability. With the addition of renewable energy sources and the advancement of smart grid technology, energy systems have become increasingly complex, making accurate forecasting increasingly challenging. Conventional techniques, including regression models and ARIMA, frequently perform less well because they are unable to capture the complex multivariate relationships and temporal dependencies present in energy data. Furthermore, these techniques are prone to errors in the presence of noisy data and have scalability issues when used on big, high-dimensional datasets. This paper presents a hybrid forecasting framework that combines artificial neural networks with Time Series Transformers and Fuzzy Logic Transform in order to overcome these drawbacks. The Transformer architecture excels in capturing long-term dependencies and interdependencies between features through its self-attention mechanism. Meanwhile, FLT + ANN effectively preprocesses noisy, irregular data and models short-term nonlinear patterns. The combination of these techniques creates a robust framework capable of handling complex energy datasets while maintaining high accuracy. Extensive tests on actual energy datasets show that the suggested hybrid model outperforms both conventional and stand-alone methods. With RMSE and MAE reductions of up to 15–20%, the model outperforms baseline models such as Random Forests, Decision Trees, and Linear Regression. These findings demonstrate how the suggested paradigm has the potential to transform load forecasting and enable more intelligent, effective energy systems.

## Introduction

The global energy sector is experiencing a paradigm shift driven by increasing energy consumption, the widespread integration of renewable energy sources, and the development of smart grid technologies. These advancements aim to enhance efficiency, reduce carbon emissions, and ensure energy sustainability. At the heart of this transformation lies the critical need for accurate load forecasting, a fundamental task that enables energy providers to anticipate demand, optimize resource allocation, reduce operational costs, and maintain grid stability. In addition to being necessary for effective grid operations, load forecasting is also critical for facilitating the smooth integration of intermittent and variable renewable energy sources like solar and wind^1^.

Load forecasting, however, is fraught with challenges due to the dynamic and complex nature of energy consumption data. Multiple factors contribute to this complexity, including nonlinear usage patterns, seasonal fluctuations, external influences like weather and holidays, and the interdependencies among various energy categories. For load forecasting, traditional statistical techniques including regression-based methods and AutoRegressive Integrated Moving Average (ARIMA) models have been used extensively. While these methods provide satisfactory results in relatively simple scenarios, they are fundamentally limited in their ability to model nonlinear trends and multivariate dependencies^2^. Moreover, their reliance on stationarity assumptions and their inability to handle large, high-dimensional datasets make them unsuitable for modern energy systems.

The advancement of machine learning has introduced novel approaches to overcoming the limitations inherent in conventional models. Recurrent neural networks (RNNs) and long short-term memory (LSTM) networks have gained popularity due to their ability to represent sequential input and capture temporal relationships^3^. These systems do, however, have a number of difficulties, especially when handling noisy datasets, high-dimensional inputs, and long-term relationships. RNNs and LSTMs are computationally costly and less scalable for large-scale applications due to their sequential structure, which restricts their applicability in energy forecasting tasks.

Transformers were first created for jobs involving natural language processing, but they have recently shown incredible promise in time series forecasting^4^. Transformers are more computationally efficient and scalable than RNNs and LSTMs because they employ a self-attention mechanism that enables them to process entire sequences in parallel. They are ideal for high-dimensional datasets because they are excellent at capturing multivariate interactions and long-term temporal dependencies. Transformers, however, are highly dependent on clean and organized data, which may impair their performance in real-world situations where noise and inconsistencies are common.

On the other hand, methods based on Fuzzy Logic Transform (FLT) combined with ANN have proven effective in handling noisy and ambiguous datasets^5^. FLT preprocesses data by converting continuous variables into fuzzy sets, simplifying noisy information and retaining critical patterns. ANN then learns nonlinear dependencies, enabling effective modeling of localized trends and short-term variations. Despite their robustness to noise, FLT + ANN approaches lack the ability to model long-term dependencies and global patterns, which are essential for accurate load forecasting in complex energy systems^6^.

To address the multifaceted challenges associated with modern energy systems, including data irregularities, nonlinear consumption patterns, temporal complexity, and high dimensionality, this study introduces a unified hybrid forecasting framework that integrates Time Series Transformer architectures with fuzzy logic transformation and artificial neural networks. The Time Series Transformer component is employed to capture long-range temporal dependencies and intricate inter-variable relationships, providing a powerful mechanism for modeling global patterns in complex energy datasets. Simultaneously, the fuzzy logic transformation enhances data robustness by converting noisy and ambiguous inputs into interpretable fuzzy sets, which are then processed by artificial neural networks to effectively model localized, nonlinear trends and short-term variations. This integration of complementary modeling strategies enables the proposed framework to deliver high predictive accuracy, strong resilience to data imperfections, and scalability across a wide range of load forecasting scenarios. By addressing the limitations inherent in traditional statistical techniques, standalone deep learning methods, and fuzzy logic approaches, the hybrid model represents a significant advancement in the development of reliable and intelligent forecasting systems for modern energy infrastructures.

The effectiveness of the hybrid model is evaluated on a comprehensive real-world energy dataset that includes hourly energy consumption data for variables such as *Cooling: Electricity*, *Heating: Gas*, and *Fans: Electricity*. Temporal features such as *Hour* and *DayOfWeek*, as well as external factors like weather conditions, are incorporated to enhance predictive accuracy. Experimental results demonstrate that the hybrid model significantly outperforms both traditional statistical methods and standalone machine learning approaches, achieving substantial reductions in RMSE, MAE, and MAPE. The hybrid framework achieves accuracy improvements compared to baseline models like Linear Regression, Decision Trees, and Random Forests.

The contributions of this work are as follows:

1. Proposes a unified hybrid model combining FLT, ANN, and Time Series Transformers to address noise, nonlinearity, and temporal dependencies in energy data.
2. Introduces an attention mechanism between raw and fuzzy features with a parallel-sequential learning strategy.
3. Validates the model on real-world NY and TX datasets, demonstrating superior forecasting performance.
4. Offers a scalable and interpretable solution to support smart grid operations and renewable energy integration.

In summary, this study presents a significant advancement in the field of load forecasting by combining state-of-the-art machine learning techniques with robust noise handling methods. The proposed hybrid framework not only delivers high-accuracy predictions, but also addresses key challenges associated with scalability and noise in energy data, paving the way for smarter and more efficient energy management systems.

The rest of the paper is organized as follows: Section “Related work” reviews related work in load forecasting. Section “Proposed methodology” presents the proposed hybrid model combining ANN, FLT, and Time Series Transformer. Section “Experimental results and analysis” details experimental results and analysis. Section “Conclusion” concludes the paper with key findings and future directions.

## Related work

Over the past few decades, load forecasting has been the subject of much research, with methodologies ranging from sophisticated machine learning and deep learning approaches to traditional statistical approaches. Depending on the type and complexity of the dataset, each of these approaches offers advantages and disadvantages.

### Traditional statistical techniques

Statistical methods such as the integrated autoregressive moving average (ARIMA), the seasonal decomposition of time series (STL), and regression models have been widely used for load forecasting due to their interpretability and ease of use. Regression models are excellent at addressing linear relationships, but ARIMA, for instance, works well for short-term forecasts in stationary time series data. Nevertheless, these approaches have inherent limitations when it comes to modeling multivariate interactions, long-term dependencies, and nonlinear patterns. Moreover, their reliance on stationarity assumptions makes them unsuitable for dynamic, high-dimensional energy datasets^7,8^.

### Machine learning approaches

The area of load forecasting has made tremendous strides with the introduction of machine learning. Complex, nonlinear interactions have been modeled using algorithms like Random Forests, Decision Trees, and Support Vector Machines (SVMs). These methods offer better accuracy compared to traditional statistical approaches, but often lack the ability to capture temporal dependencies effectively, making them less suitable for sequential data^9^.

Some of these limitations have been addressed by deep learning models such as RNNs and LSTM networks, which explicitly describe temporal correlations^10^. In identifying short- and medium-term dependencies in sequential data, LSTMs in particular have demonstrated potential. However, they are computationally costly and have limited scalability for large datasets because of their sequential processing structure. Furthermore, both RNNs and LSTMs require extensive hyperparameter tuning, which can be a barrier to widespread adoption^11,12^.

### Transformer-based models

Originally created for applications that involve natural language processing, transformers have recently become popular in time-series forecasting. In many situations, they outperform conventional RNN and LSTM models thanks to their self-attention mechanism, which allows them to capture global feature interactions and long-term dependencies. Unlike RNNs, Transformers process entire sequences in parallel, making them computationally efficient and scalable. However, their reliance on clean, structured data reduces their robustness in noisy environments, a common characteristic of real-world energy datasets^13,14^.

Several studies have explored the use of Transformers for energy forecasting. For instance, models like Informer and Time Series Transformers have demonstrated improved scalability and accuracy over conventional methods^7,15^. While these advancements address some challenges, their sensitivity to noise and lack of built-in mechanisms for handling irregular data remain significant drawbacks^16^.

### Fuzzy logic and neural network approaches

Fuzzy Logic Transform combined with Artificial Neural Networks represents another promising direction for load forecasting. FLT preprocesses raw data by converting continuous variables into fuzzy sets, simplifying the representation of noisy and ambiguous data. ANN then models nonlinear dependencies, making FLT + ANN particularly effective for noisy and irregular datasets^17^. Studies have shown that FLT + ANN outperforms traditional models in scenarios with high noise levels and complex relationships. However, FLT + ANN lacks the ability to model long-term temporal dependencies, limiting its applicability for large-scale energy forecasting^8^.

The author in^18^ applied a Long Short-Term Memory (LSTM) recurrent neural network for short-term residential load forecasting, demonstrating improved performance over conventional models such as ARIMA and feedforward neural networks. The LSTM was effective in modeling temporal dependencies in household consumption data. However, their approach did not explore newer deep learning architectures or incorporate contextual features such as weather or occupancy data. Furthermore, scalability and interpretability remained significant challenges, as separate models were required per household, and the reasoning behind predictions was not transparent.

The Deep-Autoformer architecture proposed in^19^ is tailored for very short-term load forecasting. By leveraging auto-correlation mechanisms and deep temporal feature extraction, the model achieved superior accuracy on short forecasting intervals using the Pecan Street dataset. However, their model performance degraded when longer historical sequences were included, highlighting limitations in generalizing across varying temporal contexts. Additionally, the reliance on data-specific periodicity raises concerns regarding the model’s robustness across heterogeneous user behaviors.

A spatial-temporal residential short-term load forecasting framework using Graph Neural Networks (GNNs) proposed in^20^ to model spatial correlations among households and temporal patterns in electricity consumption. Their approach effectively captures inter-household dependencies by constructing a graph structure based on physical proximity and consumption similarity, which is then combined with temporal learning to improve forecast accuracy. While the GNN-based model achieves improved performance over traditional and standalone temporal models, it relies heavily on accurately defined graph structures to model spatial relationships. This dependency on graph topology can be a limitation in scenarios where such spatial connectivity is not well-defined, dynamic, or unavailable.

The authors in^21^ advanced the field by modeling the conditional probability density function (PDF) of residential load using a deep learning framework. Their probabilistic model captures the uncertainty of future load profiles more effectively than point forecasts. However, the method assumes a fixed output distribution and requires complex training to balance likelihood estimation and regularization. Moreover, while it offers probabilistic insights, it does not directly optimize for deterministic forecasting accuracy, which is crucial in real-time applications. A recent study^22^ proposed a spatiotemporal graph attention-enabled Transformer model designed for short-term, multi-resident, multi-step residential load forecasting. The approach effectively captures both dynamic spatial and nonlinear temporal dependencies through a gated fusion mechanism and attention-based graph structure embedded within a Seq2Seq Transformer framework. However, the reliance on complex attention modules and dynamic graph construction increases computational overhead, which limits scalability.

### Hybrid models

Hybrid approaches combining multiple methods have emerged to address the limitations of standalone models^23^. For instance, models combining ARIMA with machine learning algorithms have shown improved accuracy by leveraging the strengths of both techniques^9^. Similarly, hybrid frameworks that integrate LSTMs with external preprocessing methods, such as wavelet transforms, have demonstrated better performance in capturing both short-term fluctuations and long-term trends^11^. However, such combinations often suffer from increased computational complexity and a lack of scalability.

Building upon these studies, this work introduces a hybrid framework that integrates Time Series Transformers and FLT + ANN, inspired by the strengths of hybrid models discussed in^8,11^. Unlike traditional hybrid models^24^, the proposed framework combines the scalability and long-term dependency modeling capabilities of Transformers with the noise-handling and short-term pattern recognition strengths of FLT + ANN. By addressing the limitations of standalone approaches, this work advances load forecasting methodologies, providing a robust and accurate solution for real-world energy systems. As detailed in Table 1, existing approaches struggle to balance noise robustness, computational efficiency, and temporal modeling, or significantly increase complexity without achieving consistent forecast improvements. To overcome these challenges, this study proposes a hybrid framework that combines Transformer-based temporal modeling, fuzzy logic-based noise resilience, and neural network-driven nonlinear feature learning for scalable and accurate load forecasting.

**Table 1:** Comprehensive comparison of existing load forecasting models.

Method	Dataset used	Metrics used	Strengths	Limitations
Traditional statistical techniques				
ARIMA, Regression, STL^7^	Time series load data	RMSE, MAE	Easy to implement, interpretable	Poor with nonlinearity, high dimensions
Machine learning approaches				
SVM, Decision Trees, RF^3^	Energy consumption datasets	Accuracy, RMSE, MAPE	Good for low-dimensional patterns	Fails on temporal patterns
Spatiotemporal GNN^11^	Multi-area energy systems	RMSE, MAPE	Models spatiotemporal dependencies	Depends on static graph design
Transformer-based models				
TS2ARCformer (Transformer variant)^4^	Multi-dimensional time series	MAPE, RMSE	Captures multivariate interactions	Needs clean data, less robust to noise
Spatiotemporal Graph Attention Transformer^13^	Multi-resident load dataset	MAPE, MAE, RMSE	Models spatial and temporal dynamics	High computational cost; low scalability
Fuzzy logic and neural network approaches				
FLT + ANN^5^	Noisy residential load data	RMSE, MAPE	Handles noise and fuzzy inputs	Limited long-term temporal modeling
Fuzzy Logic-based Preprocessing^17^	Simulated/real-world fuzzy inputs	Accuracy, MAE	Improves pattern recognition in noisy data	Limited scalability
Hybrid models				
ARIMA + Ensemble Learning^23^	Integrated energy system	MAPE, RMSE	Combines linear + nonlinear traits	May overfit; lacks temporal modeling
Neural Network + Evolutionary Optimization^8^	Short-term power grid data	MAPE, RMSE, MAE	Optimizes NN via metaheuristics	Computational overhead, hard tuning
Transformer + FLT^6^	Energy data (multi-regional)	MAPE, RMSE	Captures both fuzzy and long-term patterns	Model complexity and training time
Recent deep learning models				
LSTM^18^	Residential household consumption (HK)	MAPE, RMSE	Captures temporal dependencies	No spatial/contextual data; low interpretability
Deep-autoformer^19^	Pecan Street (Very short-term)	MAPE, MAE	Uses auto-correlation for short-term	Performance degrades on longer sequences
Graph Neural Network (GNN)^20^	Residential load with spatial graph	MAPE, RMSE	Captures spatial and temporal features	Requires static and accurate graph topology
Probabilistic Deep Learning^21^	Residential loads (PDF modeling)	MAE, PDF accuracy	Captures uncertainty via PDF	Assumes fixed distribution; complex training

## Proposed methodology

The proposed methodology combines an advanced preprocessing pipeline with a robust Time Series Transformer architecture to deliver accurate and scalable energy forecasting. While the Time Series Transformer uses its design to capture complicated temporal patterns and long-term dependencies in the data, the preprocessing procedures guarantee data quality, identify pertinent features, and get the dataset ready for efficient training. These components work in tandem to address challenges like noise, seasonality, and temporal interdependencies in energy datasets.

### Dataset description

The dataset consists of hourly measurements of energy consumption, spanning an entire year, with several temporal and external variables included to capture complex seasonal and temporal patterns. The features in the dataset include Electricity: Facility [kW], which represents the total electricity consumption of the entire facility, encompassing HVAC systems, lighting, appliances, and other electrical systems, measured in kilowatts (kW) on an hourly basis. Similarly, Gas: Facility [kW] denotes the total gas consumption by the facility, typically used for heating purposes or other gas-powered systems, with the measurement converted to energy equivalents in kilowatts. The Heating: Gas [kW] column captures gas consumption specifically for space heating systems, such as boilers or furnaces, while Cooling: Electricity [kW] records the electricity usage of cooling systems, including air conditioning or chillers, on an hourly basis. The dataset also includes HVACFan: Fans: Electricity [kW], representing the electricity consumed by HVAC fans that regulate airflow and maintain air circulation, and Electricity: HVAC [kW], which tracks the overall electricity usage of HVAC systems, encompassing both cooling and heating components as well as ventilation.

Additional features include Fans: Electricity [kW], which measures electricity consumed by fans used for air distribution within the facility, and General: InteriorLights: Electricity [kW] and General: ExteriorLights: Electricity [kW], which represent the energy usage of interior and exterior lighting systems, respectively. The dataset also captures Appl: InteriorEquipment: Electricity [kW], which includes the energy consumed by appliances such as refrigerators, computers, or microwaves, and Misc: InteriorEquipment: Electricity [kW], which covers miscellaneous devices like printers and coffee machines. The Water Heater: WaterSystems: Electricity [kW] feature represents the electricity used by water heating systems, commonly employed for hot water supply in kitchens or bathrooms.

**Fig. 1:**
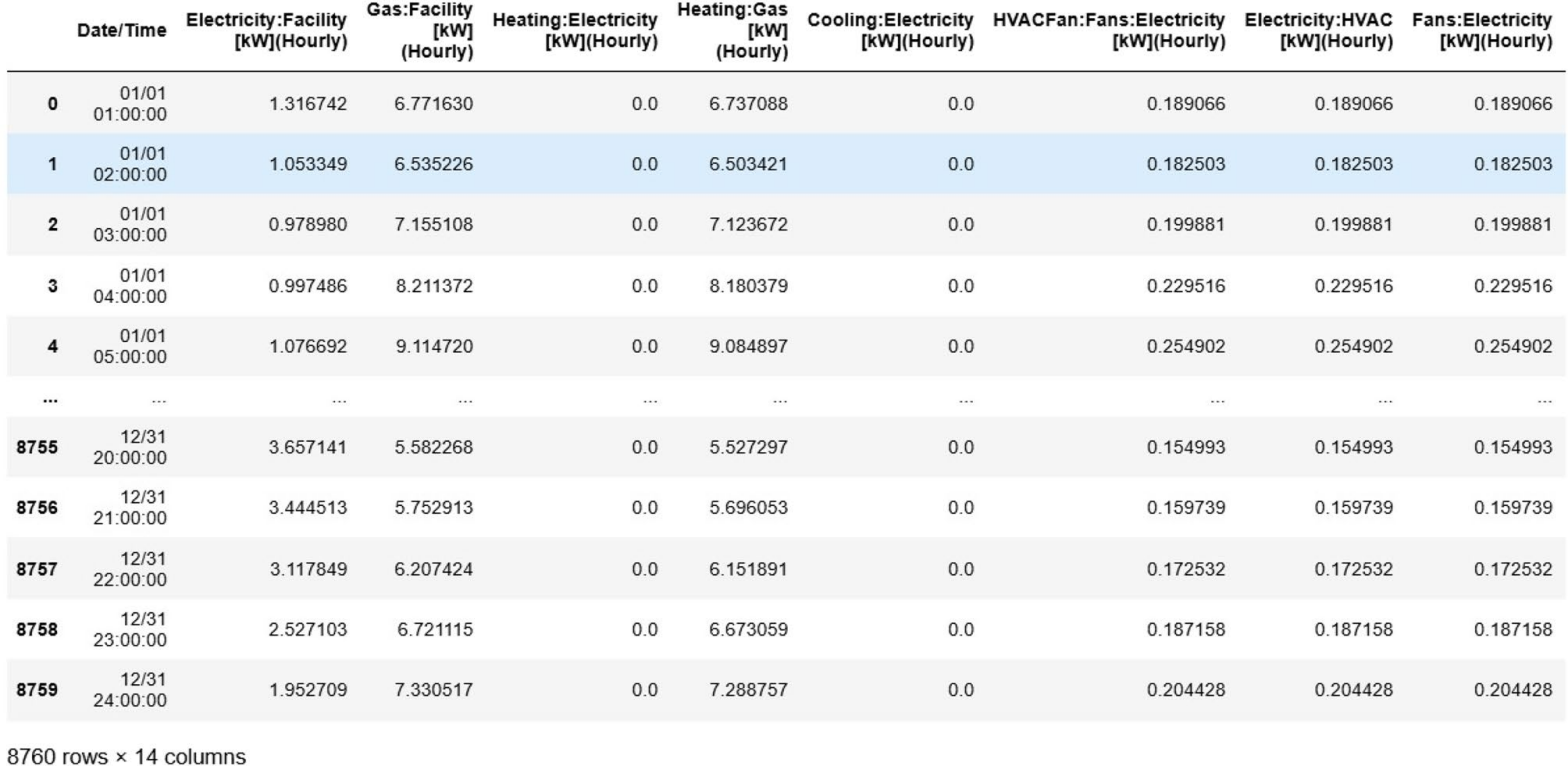
Sample dataset showcasing energy consumption records with relevant temporal variables.

The temporal variables include Hour, which indicates the specific hour of the day (ranging from 1 to 24), DayOfWeek, which represents the day of the week (1 for Monday to 7 for Sunday), and Month, which captures the month of the year (1 for January to 12 for December). These variables are crucial for identifying time-dependent energy consumption patterns, such as higher electricity demand during summer months for cooling or increased gas consumption in winter for heating. A sample dataset showcasing these energy consumption records with the relevant temporal variables is presented in Fig. 1.

The efficacy of the suggested approach is verified using publicly accessible datasets from three different areas. These datasets were gathered from two publicly accessible websites. OpenEI Residential Load Data: Hourly residential load demands from several US cities and states are included in this dataset. For this study, one-year load data from Birmingham, Alabama in 2012 was selected. The dataset comprises detailed energy usage information from residential facilities, including heating, cooling, lighting, and appliances, allowing for a comprehensive analysis of energy consumption trends.

The detailed information regarding the dataset is given in^25^: OpenEI Residential Load Data, ”Residential Load Data E Plus Output (High).”

### Preprocessing

The preprocessing phase plays a crucial role in ensuring the dataset is clean, structured, and ready for modeling. The first step involves data cleaning, where irrelevant features, such as *Heating:Electricity [kW](Hourly)*, are removed to focus on relevant predictors. Timestamp anomalies, like entries with *24:00:00*, are corrected by converting them to *00:00:00* on the following day, ensuring a uniform and interpretable temporal format. The corrected timestamps are standardized into a consistent *YYYY-MM-DD HH:MM:SS* format, which is crucial for accurate feature extraction.

Temporal features such as Hour, Day of the Week, and Month are engineered to capture cyclical and seasonal trends in energy consumption, which are significant for forecasting. The input and target variables are scaled to a range of $[0,1]$ using Min-Max Scaling, ensuring uniform contribution of all features during training. In order to provide enough training data and save a representative test set for assessment, the data is divided into training and testing sets in an 80:20 ratio. Finally, the data is organized into mini-batches for efficient gradient-based optimization, enhancing model training by allowing parallel processing and reducing computation time.

### Model 1: time series transformer

The core of the suggested forecasting methodology is the Time Series Transformer, which was created especially to get beyond traditional models’ inability to capture the intricate temporal patterns and long-term interdependence present in time-series data^4,26^. By adding techniques to handle the particular difficulties of sequential energy data, its creative architecture utilizes the power of transformers-which were first created for natural language processing-for time-series forecasting.

**Fig. 2:**
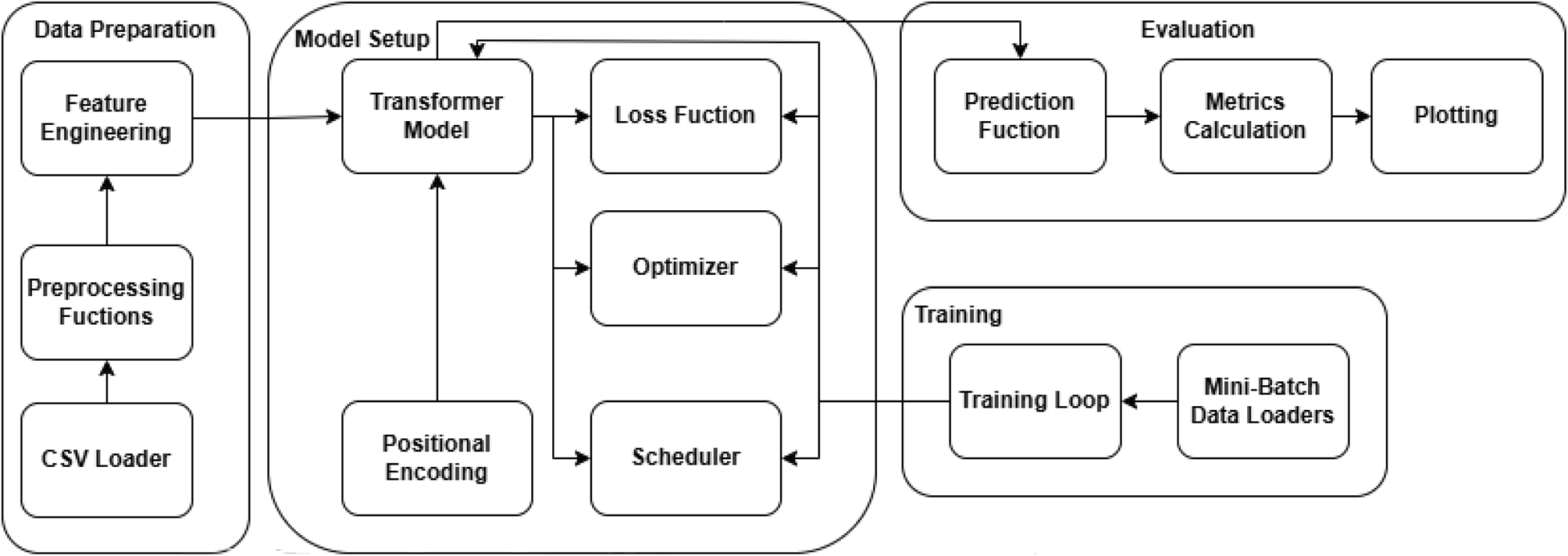
Time Series Transformer architecture illustrating the embedding, self-attention, and feedforward layers, designed to capture intricate temporal patterns in energy forecasting.

#### Input embedding and positional encoding

In order to properly depict complex interactions between variables, the model first embeds the input characteristics into a higher-dimensional space. This embedding process translates raw input data into a more abstract and rich feature representation, crucial for capturing complex dependencies.

Recognizing the sequential nature of time-series data, positional encoding is added to these embeddings, ensuring the model retains awareness of the temporal ordering of input data points. The positional encoding is defined as Eqs. (1)–(2):

1 $$\begin{aligned} PE_{(pos, 2i)} = \sin \left( \frac{pos}{10000^{\frac{2i}{d_{\text {model}}}}}\right) \end{aligned}$$

2 $$\begin{aligned} PE_{(pos, 2i+1)} = \cos \left( \frac{pos}{10000^{\frac{2i}{d_{\text {model}}}}}\right) \end{aligned}$$

where $pos$ is the position, $i$ is the dimension index, and $d_{\text {model}}$ is the embedding dimension. This ensures the model understands the significance of temporal progression, which is essential for accurate forecasting in scenarios where the timing of events influences their relevance.

#### Self-attention and multi-head attention mechanisms

The central self-attention mechanism of the Time Series Transformer dynamically determines and allocates weights to the input sequence’s most pertinent time steps^27^. This mechanism operates as in (3):

3 $$\begin{aligned} \text {Attention}(Q, K, V) = \text {softmax}\left( \frac{QK^T}{\sqrt{d_k}}\right) V \end{aligned}$$

where $Q$, $K$, and $V$ are the query, key, and value matrices, respectively, and $d_k$ is the dimension of the keys.

This ability is expanded by multi-head attention, which processes information in several subspaces at once. Each attention head’s output is concatenated and subjected to a linear transformation as outlined in Eq. (4):

4 $$\begin{aligned} \text {MultiHead}(Q, K, V) = \text {Concat}\left( \text {head}_1, \dots , \text {head}_h\right) W^O, \end{aligned}$$

where each $\text {head}_i = \text {Attention}(QW_i^Q, KW_i^K, VW_i^V)$, and $W^O$ is the output weight matrix. This allows the model to capture diverse patterns across different subspaces.

#### Feedforward neural network and regularization techniques

The outputs of the attention layers are passed through a feedforward neural network, which enhances the model’s ability to capture nonlinear relationships among features. This network is typically a two-layer MLP as follows:

5 $$\begin{aligned} \text {FFN}(x) = \max (0, xW_1 + b_1)W_2 + b_2, \end{aligned}$$

where $W_1$, $W_2$, $b_1$, and $b_2$ are the weights and biases of the layers, and $\max (0, x)$ denotes the ReLU activation function.

Regularization strategies like dropout and layer normalization are used to keep the model from overfitting and enhance its capacity for generalization. Dropout randomly disables a subset of neurons during training, reducing reliance on specific pathways, and layer normalization stabilizes training by normalizing inputs across the layers as given in Eq. (6):

6 $$\begin{aligned} \text {LayerNorm}(x) = \frac{x - \mu }{\sigma } \cdot \gamma + \beta , \end{aligned}$$

where $\mu$ and $\sigma$ are the mean and standard deviation of the input $x$, and $\gamma$ and $\beta$ are learnable parameters.

#### Optimization and training dynamics

The training process for the Time Series Transformer is carefully designed to maximize its predictive performance^28,29^. The difference between expected and actual values is measured using the Mean Squared Error (MSE) loss function, which is defined as given in (7):

7 $$\begin{aligned} \text {MSE} = \frac{1}{N} \sum _{i=1}^N (y_i - {\hat{y}}_i)^2, \end{aligned}$$

where $N$ is the number of samples, $y_i$ is the true value, and ${\hat{y}}_i$ is the predicted value.

The AdamW optimizer, which combines weight decay and adaptive learning rate techniques for improved generalization and effective convergence, is utilized to optimize the training process. The AdamW update rule is provided in (8).

8 $$\begin{aligned} \theta _{t+1} = \theta _t - \eta \cdot \frac{m_t}{\sqrt{v_t} + \epsilon } - \eta \cdot \lambda \cdot \theta _t, \end{aligned}$$

where $\eta$ is the learning rate, $m_t$ and $v_t$ are the moving averages of the gradient and squared gradient, $\epsilon$ is a small constant for numerical stability, and $\lambda$ is the weight decay coefficient.

Furthermore, to dynamically modify the learning rate during training, a cosine annealing learning rate scheduler is used. This scheduler cyclically lowers the learning rate over time as outlined in (9).

9 $$\begin{aligned} \eta _t = \eta _{\text {min}} + \frac{1}{2}(\eta _{\text {max}} - \eta _{\text {min}})\left( 1 + \cos \left( \frac{t}{T_{\text {max}}}\pi \right) \right) , \end{aligned}$$

where $\eta _{\text {min}}$ and $\eta _{\text {max}}$ are the minimum and maximum learning rates, and $T_{\text {max}}$ is the maximum number of iterations. The overall detailed steps processed in the time series transformer are shown in Fig. 2.

#### Integration and real-world applicability

By combining a rigorous preprocessing pipeline with the sophisticated Time Series Transformer architecture, the proposed methodology effectively addresses key challenges such as noise, seasonality, and variability in energy consumption data. The transformer’s ability to model intricate temporal dependencies, identify nonlinear relationships, and generalize to unseen patterns makes it a robust and scalable solution for energy forecasting. Its performance in handling complex datasets underscores its potential for deployment in real-world energy management systems, enabling better decision-making and resource optimization.

### Model 2: artificial neural network with fuzzy logic transformation (ANN+FLT)

The Artificial Neural Network combined with Fuzzy Logic Transformation forms a hybrid architecture tailored to improve the modeling of energy consumption data. This innovative approach leverages FLT to preprocess noisy and ambiguous data and ANN’s ability to model nonlinear relationships, enabling accurate and interpretable energy forecasting.

#### Input embedding and fuzzy logic transformation

In the ANN+FLT methodology, input data undergoes Fuzzy Logic Transformation to enhance feature representation^24,30^. FLT applies a set of predefined rules to process raw continuous variables into fuzzy sets. These transformations include:

- *Normalization:* Ensures the data is scaled to a uniform range, reducing the effect of outliers and enabling consistent input for the neural network. This is achieved using Eq. (10).
  10 $$\begin{aligned} \text {Normalization Rule: } f(x) = \frac{x^2}{1 + x^2} \end{aligned}$$
- *Mean-Centering:* Highlights deviations from the mean, making patterns within the data more discernible. The mean-centering transformation is defined in Eq. (11).
  11 $$\begin{aligned} \text {Mean-Centering Rule: } f(x) = x - \mu , \quad \mu = \frac{1}{n} \sum _{i=1}^n x_i \end{aligned}$$
- *Nonlinear Transformation (Sigmoid):* Compresses values into a bounded range, emphasizing differences in smaller input variations. The Eq. (12) explains the nonlinear transformation rule as follows:
  12 $$\begin{aligned} \text {Nonlinear Transformation Rule: } f(x) = \frac{1}{1 + e^{-x}} \end{aligned}$$

This preprocessing stage simplifies complex patterns and reduces noise, offering a complementary representation to the raw features. The transformed features, highlighting key localized and global patterns, are then passed into the neural network for further processing.

#### ANN + FLT architecture

**Fig. 3:**
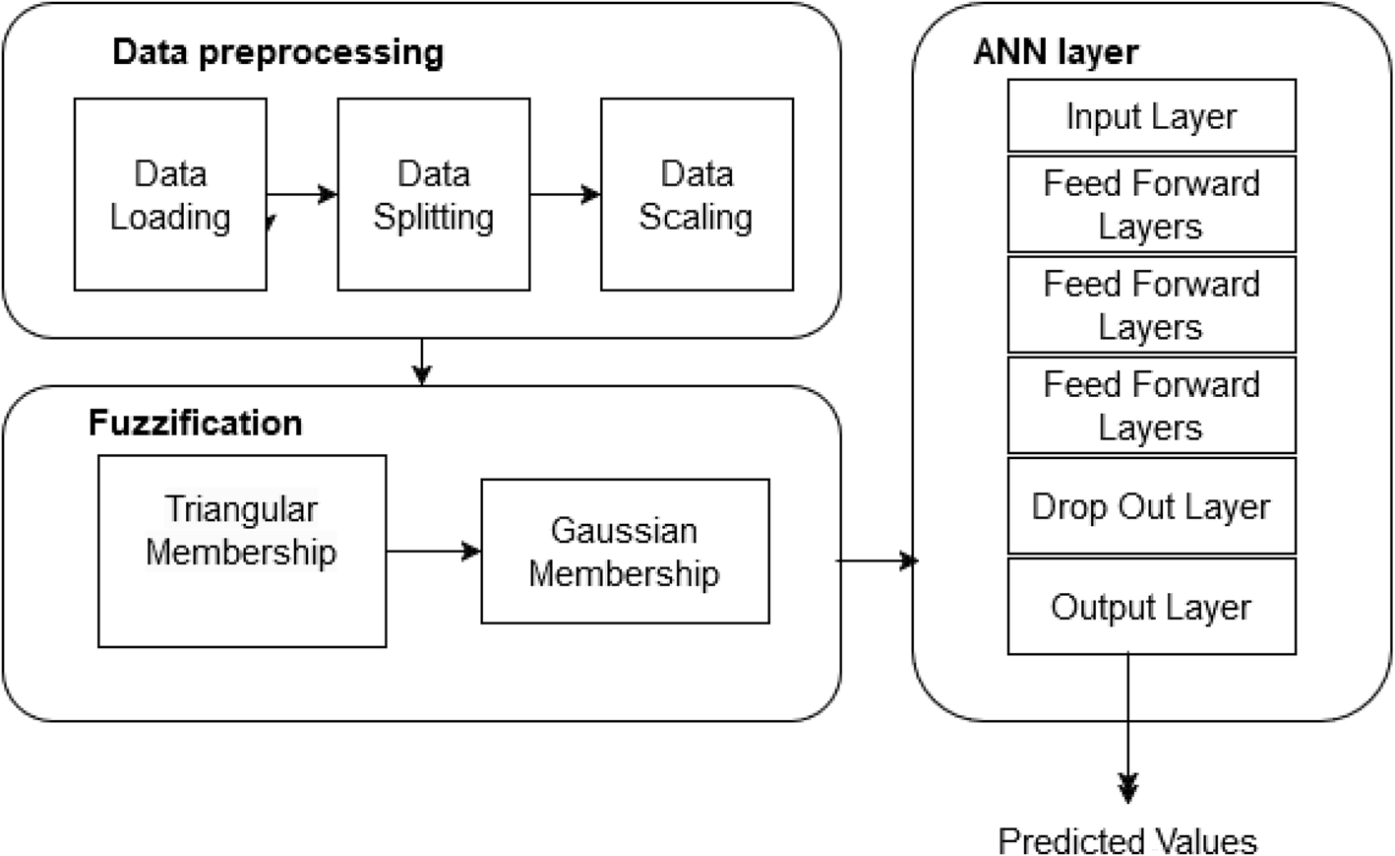
The ANN+FLT hybrid model architecture, illustrating the dual-path structure for raw and fuzzy-transformed features, attention mechanism, and subsequent processing.

The architecture of the ANN + FLT model is shown in Fig. 3 illustrating the dual path structure for the raw and fuzzy transformed features, the attention mechanism, and the subsequent processing.

1. *Raw Feature Path:* Batch normalization, dropout algorithms, and a sequence of fully linked layers are used to process the raw input data. This path captures the nonlinear relationships and long-term dependencies within the data.
2. *Fuzzy-Transformed Path:* Features transformed through FLT are processed separately, allowing the network to extract additional insights from the enhanced representation of noisy data.

The outputs from these paths are concatenated and passed through an attention mechanism, which dynamically assigns weights to the raw and fuzzy-transformed features. This mechanism ensures the model adapts its focus based on the relevance of each feature type, enabling it to learn robust and generalized patterns from both inputs.

#### Feedforward neural network and regularization techniques

Each path processes the inputs through multiple hidden layers, reducing dimensionality and extracting meaningful representations. The concatenated features are further refined through additional feedforward layers, capturing complex interactions between the raw and fuzzy-transformed features.

To enhance generalization and prevent overfitting, the model employs dropout layers that randomly deactivate neurons during training, reducing over-dependence on specific pathways. Batch normalization stabilizes the learning process by normalizing activations, ensuring consistent training dynamics even in the presence of noisy data.

#### Optimization and training dynamics

The training process for the ANN+FLT model is optimized to ensure reliable convergence and accurate predictions. The following techniques are employed:

- *Loss Function:* The Mean Squared Error (MSE) is used as the loss function, as defined earlier in Eq. (7).
- *Optimizer:* The AdamW optimizer combines adaptive learning rates with weight decay for improved generalization as given in Eq. (14).
  13 $$\begin{aligned} \theta _{t+1} = \theta _t - \eta \cdot \frac{m_t}{\sqrt{v_t} + \epsilon } + \lambda \cdot \theta _t \end{aligned}$$
  where $m_t$ and $v_t$ are the first and second moment estimates, and $\lambda$ is the weight decay coefficient.
- *Learning Rate Scheduler:* Based on validation performance, a ReduceLROnPlateau scheduler dynamically modifies the learning rate.
- *Early Stopping:* Monitors the validation loss to halt training once performance stagnates.

#### Integration and real-world applicability

The ANN + FLT hybrid architecture offers a robust solution for energy forecasting by combining the strengths of fuzzy logic and neural networks. FLT simplifies noisy and ambiguous patterns, making them more interpretable, while the ANN captures complex nonlinear relationships within the data. This integration makes the model particularly suited for datasets characterized by noise, short-term variations, and localized patterns, as is common in energy consumption data.

By leveraging fuzzy-transformed features alongside raw inputs, the model achieves superior performance in capturing both global trends and localized dependencies. This hybrid approach enables it to deliver accurate, scalable, and interpretable solutions for real-world energy management systems, supporting decision-making in dynamic and complex environments.

### Model 3: hybrid model: ANN + FLT + time series transformer

**Fig. 4:**
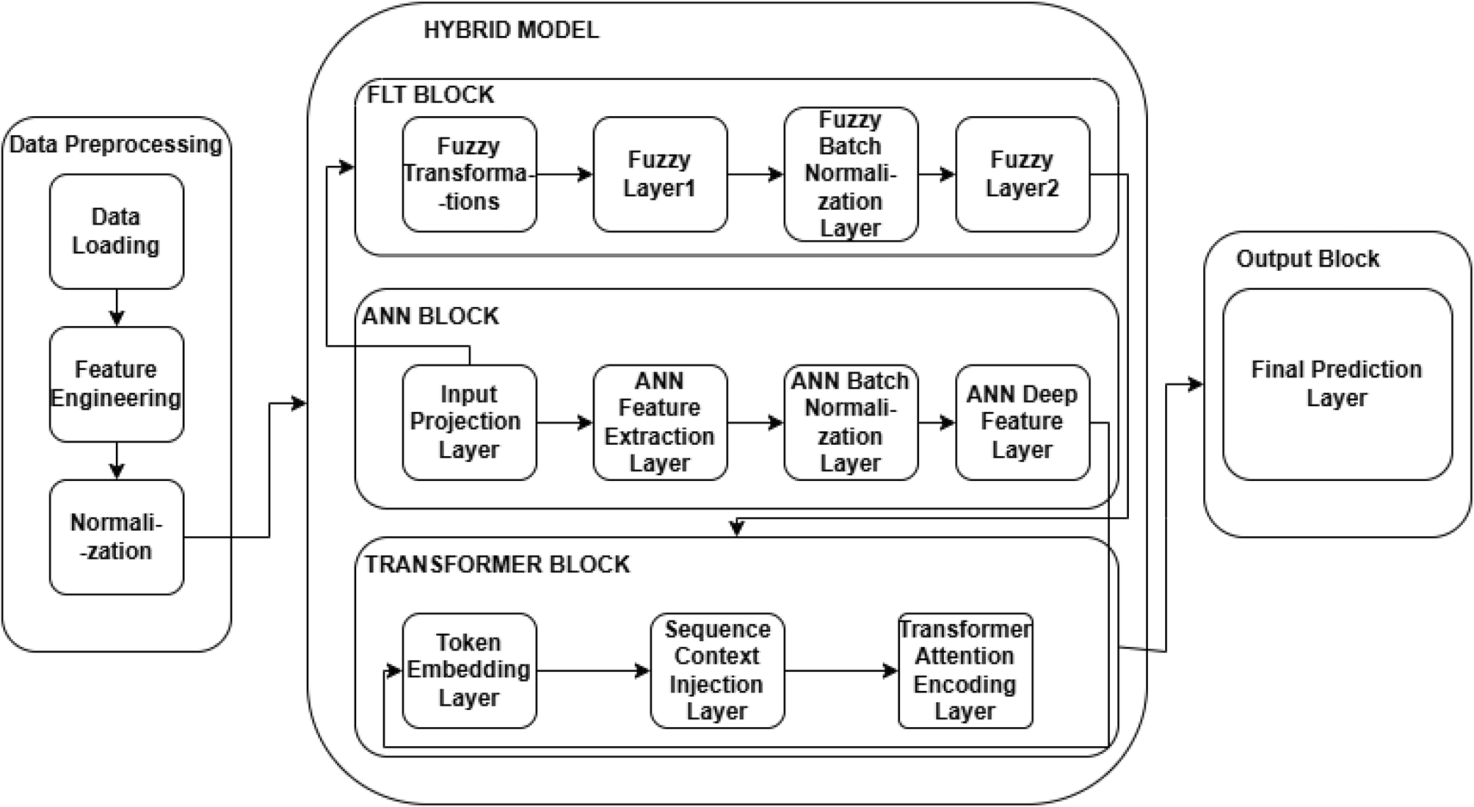
Hybrid model Architecture of the ANN with Fuzzy Logic Transformation and Time Series Transformation.

This hybrid model integrates Artificial Neural Networks, Fuzzy Logic Transformation, and Time Series Transformers to predict energy consumption, as depicted in Fig. 4. The model leverages the feature extraction and nonlinear learning capabilities of ANN, the enhanced feature representation provided by FLT, and the temporal dependency handling of the Transformer architecture.

#### Data preprocessing and feature engineering

The dataset is first preprocessed to remove unnecessary columns and to correct time format issues. The relevant columns are then extracted and features are derived from the Date/Time field as follows:

$$\begin{aligned} & \text {Hour} = \text {Hour of the day from Date/Time} \\ & \text {DayOfWeek} = \text {Day of the week from Date/Time} \\ & \text {Month} = \text {Month of the year from Date/Time} \end{aligned}$$

The preprocessing ensures all time-dependent features are extracted and normalized, facilitating consistent training of the model.

#### Normalization and scaling

Normalization is applied to the input and target variables using Min-Max scaling, ensuring that the data is scaled to the range $[0, 1]$. The normalization formula is as follows:

14 $$\begin{aligned} X_{\text {norm}} = \frac{X - \min (X)}{\max (X) - \min (X)} \end{aligned}$$

15 $$\begin{aligned} Y_{\text {norm}} = \frac{Y - \min (Y)}{\max (Y) - \min (Y)} \end{aligned}$$

where $X$ and $Y$ are the original input and target values, and $X_{\text {norm}}$ and $Y_{\text {norm}}$ are the normalized values.

#### Model architecture: ANN + FLT + transformer

The hybrid model architecture combines three powerful components: the Artificial Neural Network, Fuzzy Logic Transformation, and Time Series Transformer. As shown in Fig. 4 Each component plays a crucial role in capturing different aspects of the data, ensuring accurate predictions by leveraging both local and global dependencies, as well as nonlinear transformations. The following subsections explain the individual components of the model in more detail.

***ANN Block*** The Artificial Neural Network block is designed to learn complex, nonlinear relationships between input features. The ReLU activation function comes after each of the two completely connected layers that make up this block. By adding nonlinearity to the model, ReLU enables it to recognize complex patterns in the data. Furthermore, the activations are standardized using batch normalization, which lowers internal covariate shift and enhances convergence during training. By randomly setting a portion of the activations to zero during training, dropout regularization is used to avoid overfitting.

The output of the ANN block is calculated as described in Eqs. (17)–(19).

16 $$\begin{aligned} x_{\text {ann}} = \text {ReLU}(W_1 x + b_1) \end{aligned}$$

17 $$\begin{aligned} x_{\text {ann}} = \text {BatchNorm}(\text {ReLU}(W_2 x_{\text {ann}} + b_2)) \end{aligned}$$

18 $$\begin{aligned} x_{\text {ann}} = \text {Dropout}(x_{\text {ann}}) \end{aligned}$$

where $W_1, W_2$ are the weight matrices, $b_1, b_2$ are the bias terms, and $x$ is the input feature vector. These operations allow the ANN block to capture the complex patterns within the input data, preparing the features for the subsequent processing stages. ***FLT Block*** The Fuzzy Logic Transformation block is inspired by fuzzy logic systems, where nonlinear transformations are applied to the input features to simulate human-like decision-making^17^. In this block, several transformations are performed on the input features, such as normalization, mean centering, and nonlinear activations. This design helps the model adapt to uncertainty, which is common in real-world data. Similar to the ANN block, the FLT block consists of fully connected layers followed by ReLU activations, batch normalization, and dropout. The output of the FLT block is computed as described in Eqs. (20)–(22) :

19 $$\begin{aligned} x_{\text {flt}} = \text {ReLU}(W_{\text {flt1}} x_{\text {flt}} + b_{\text {flt1}}) \end{aligned}$$

20 $$\begin{aligned} x_{\text {flt}} = \text {BatchNorm}(\text {ReLU}(W_{\text {flt2}} x_{\text {flt}} + b_{\text {flt2}})) \end{aligned}$$

21 $$\begin{aligned} x_{\text {flt}} = \text {Dropout}(x_{\text {flt}}) \end{aligned}$$

where $W_{\text {flt1}}, W_{\text {flt2}}$ and $b_{\text {flt1}}, b_{\text {flt2}}$ are the weight matrices and biases for the FLT layers. The FLT block makes the model more resilient to changes in the data distribution by improving its capacity to manage fuzziness and nonlinearities in the input data.

***Transformer Block***

For time series forecasting and sequence modeling tasks, the Transformer block is in charge of identifying long-range dependencies and sequential patterns in the incoming data. First, a completely linked layer is used to integrate the Transformer block’s input into a higher-dimensional space^31^. This model may then learn temporal correlations in sequential data by adding positional encodings, which provide information about the relative placements of the data points. The model can then concentrate on various sequence segments and identify long-range dependencies thanks to the Transformer’s self-attention mechanism. When modeling time series data, this is especially helpful because past events-even if they occurred a long time ago-may influence future projections.

The output of the Transformer block is computed as follows:

**Algorithm 1:**
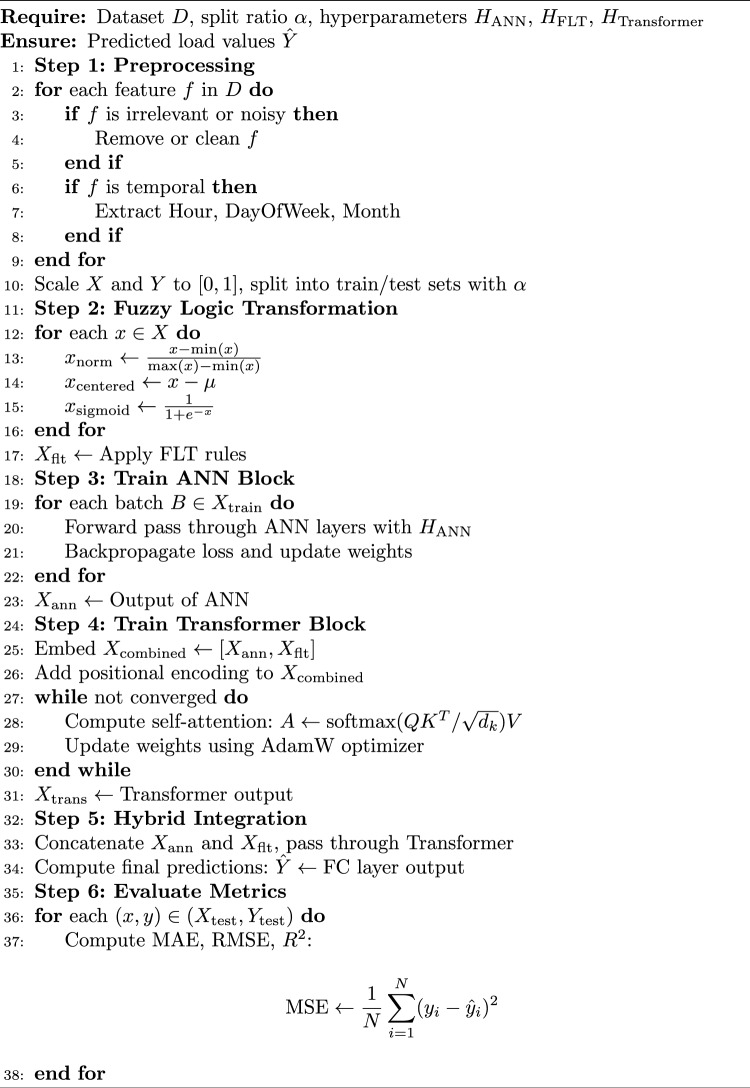
Hybrid Load Forecasting Framework

22 $$\begin{aligned} x_{\text {embed}} = W_{\text {embed}} x_{\text {combined}} \end{aligned}$$

23 $$\begin{aligned} x_{\text {pos}} = \text {PositionalEncoding}(x_{\text {embed}}) \end{aligned}$$

24 $$\begin{aligned} x_{\text {attn}} = \text {TransformerEncoder}(x_{\text {pos}}) \end{aligned}$$

where $x_{\text {combined}}$ is the combined output from both the ANN and FLT blocks, and $W_{\text {embed}}$ is the embedding matrix. The Transformer encoder’s self-attention mechanism enables the model to learn both short-term and long-term dependencies by dynamically shifting the emphasis on various input sequence segments.

***Output Block***

Finally, the output block produces the predictions by passing the output of the Transformer through a fully connected layer. This layer maps the high-dimensional representation learned by the Transformer back to the desired output space, where the predictions are made for each target variable.

The output of the model is computed as given in Eq. (26).

25 $$\begin{aligned} y_{\text {pred}} = W_{\text {out}} x_{\text {attn}} + b_{\text {out}} \end{aligned}$$

where $W_{\text {out}}$ is the output weight matrix and $b_{\text {out}}$ is the bias. The fully connected layer ensures that the model’s predictions are in the correct output format, matching the dimensionality and scale of the target variables. The detailed steps of the proposed hybrid model are outlined in Algorithm 1.

This architecture allows the model to leverage the strengths of each individual component-ANN for capturing local patterns, FLT for nonlinear transformations, and Transformer for long-range dependencies-resulting in a highly effective model for time series forecasting and other sequential tasks.

#### Training and optimization

***Training Strategy***

The model is trained using the AdamW optimizer, which updates the parameters of the model using the following rule:

26 $$\begin{aligned} \theta _t = \theta _{t-1} - \eta \frac{m_t}{\sqrt{v_t} + \epsilon } \end{aligned}$$

where $\theta _t$ represents the model’s parameters at time step $t$, $m_t$ and $v_t$ are the first and second moment estimates, $\eta$ is the learning rate, and $\epsilon$ is a small constant.

***Loss Function***

The difference between expected and actual data is calculated using the Mean Squared Error (MSE) loss function as per Eq. (28).

27 $$\begin{aligned} L = \frac{1}{N} \sum _{i=1}^{N} (y_i - {\hat{y}}_i)^2 \end{aligned}$$

where $N$ is the number of samples, $y_i$ is the true value, and ${\hat{y}}_i$ is the predicted value.

***Regularization***

Dropout regularization is applied during training with a probability $p$ of keeping each unit in the layer as represented in Eq. (29).

28 $$\begin{aligned} \text {Dropout}(x) = \text {Mask}(x, p) \end{aligned}$$

where $\text {Mask}(x, p)$ randomly drops units in the layer with probability $1-p$.

The ANN + FLT + Transformer hybrid model successfully combines the strengths of ANN, FLT, and Transformer models to provide accurate and interpretable energy consumption forecasts. The fuzzy logic transformation enhances the feature representation, while the ANN captures nonlinear relationships and the Transformer handles temporal dependencies. This makes the model effective in forecasting energy data with noise, short-term fluctuations, and long-range trends.

## Experimental results and analysis

In this section, we present and analyze the performance outcomes of the three models: the Hybrid Model, ANN + FLT, and Time Series Transformer, based on various evaluation criteria. The measurements shed light on how well the models perform in terms of model fit, error magnitude, and peak energy usage prediction.

### Experimental setup

The experimental setup was implemented in Python 3.9 on a computing system equipped with an Intel Core i7 processor and 16 GB of RAM. The experiments utilized several key libraries from the Python ecosystem to ensure efficiency, reproducibility, and scalability of the forecasting pipeline. Numerical computations and array operations were handled using NumPy, while Pandas was employed for data preprocessing and manipulation. Machine learning algorithms and performance evaluation metrics were implemented using Scikit-learn. Visualization of results and performance comparisons were carried out using Matplotlib and Seaborn. Deep learning models, including the proposed Hybrid Model and the Time Series Transformer, were developed and trained using TensorFlow. This environment facilitated robust experimentation for energy consumption forecasting, ensuring consistency and transparency in the evaluation process.

**Table 2:** Final hyperparameters used for each model.

Model	Hyperparameters
Time series transformer	Activation_function=ReLU, batch_size=32, dropout=0.3, embed_dim=128, epochs=100, ff_dim=256, learning_rate (lr)=0.001, num_heads=8, num_layers=3, optimizer=AdamW, scheduler_type=CosineAnnealingLR, weight_decay=0.01
ANN + FLT	Activation=ReLU, batch_size=64, dropout_rate=0.3, epochs=100, hidden_layers=[128, 64, 32], learning_rate (lr)=0.001, method (FLT)=Triangular, num_terms (FLT)=5, optimizer=Adam, fuzzify_strategy (FLT)=max
Hybrid ANN + FLT + Transformer	Activation=ReLU, batch_size=32, dropout (ANN block)=0.4, dropout (Transformer)=0.4, embed_dim=128, epochs=100, ff_dim=256, fuzzify_strategy (FLT)=max, hidden_size=256, input_size=3, learning_rate (lr)=0.001, method (FLT)=Triangular, num_heads=8, num_layers=3, num_terms (FLT)=5, optimizer=AdamW, output_size=10, scheduler=CosineAnnealingLR (T_max=10), weight_decay=1e-4

### Evaluation metrics

Model performance was evaluated using a range of established statistical metrics. The Root Mean Squared Error (RMSE) quantifies the average magnitude of prediction errors, placing greater emphasis on larger errors. It is defined as:

29 $$\begin{aligned} \text {RMSE} = \sqrt{\frac{1}{n} \sum _{i=1}^{n} (y_i - {\hat{y}}_i)^2} \end{aligned}$$

where $y_i$ and ${\hat{y}}_i$ denote the actual and predicted values, respectively.

The Mean Absolute Error (MAE) represents the average absolute difference between actual and predicted values, without considering the direction of the errors. It is computed as:

30 $$\begin{aligned} \text {MAE} = \frac{1}{n} \sum _{i=1}^{n} |y_i - {\hat{y}}_i| \end{aligned}$$

The Normalized Mean Squared Error (NMSE) scales the squared error relative to the actual values, offering a dimensionless metric suitable for comparisons across datasets. It is given by:

31 $$\begin{aligned} \text {NMSE} = \frac{1}{n} \sum _{i=1}^{n} \left( \frac{y_i - {\hat{y}}_i}{y_i} \right) ^2 \end{aligned}$$

The Mean Absolute Percentage Error (MAPE) expresses prediction accuracy as a percentage, which is particularly useful for comparing model performance across different scales. It is calculated as:

32 $$\begin{aligned} \text {MAPE} = \frac{1}{n} \sum _{i=1}^{n} \left| \frac{y_i - {\hat{y}}_i}{y_i} \right| \times 100 \end{aligned}$$

To assess how well the model captures peak demand, the Peak Prediction Error (PPE) was used. This metric evaluates the maximum absolute deviation between the predicted and actual peak values:

33 $$\begin{aligned} \text {PPE} = \max \left( \left| y_{\text {peak}} - {\hat{y}}_{\text {peak}} \right| \right) \end{aligned}$$

The coefficient of determination (${\textrm{R}}_{2}$) measures the proportion of variance in the actual values that can be explained by the model’s predictions. It is defined as:

34 $$\begin{aligned} R^2 = 1 - \frac{\sum _{i=1}^{n} (y_i - {\hat{y}}_i)^2}{\sum _{i=1}^{n} (y_i - {\bar{y}})^2} \end{aligned}$$

where ${\bar{y}}$ represents the mean of the actual values.

Finally, Accuracy reflects the percentage of predictions that match the rounded true values. It is expressed as:

35 $$\begin{aligned} \text {Accuracy} = \left( \frac{\sum _{i=1}^{n} {\mathbb {I}}\left( \text {round}(y_i) = \text {round}({\hat{y}}_i)\right) }{n} \right) \times 100 \end{aligned}$$

where ${\mathbb {I}}(\cdot )$ is the indicator function that returns 1 if the condition is true and 0 otherwise.

**Fig. 5:**
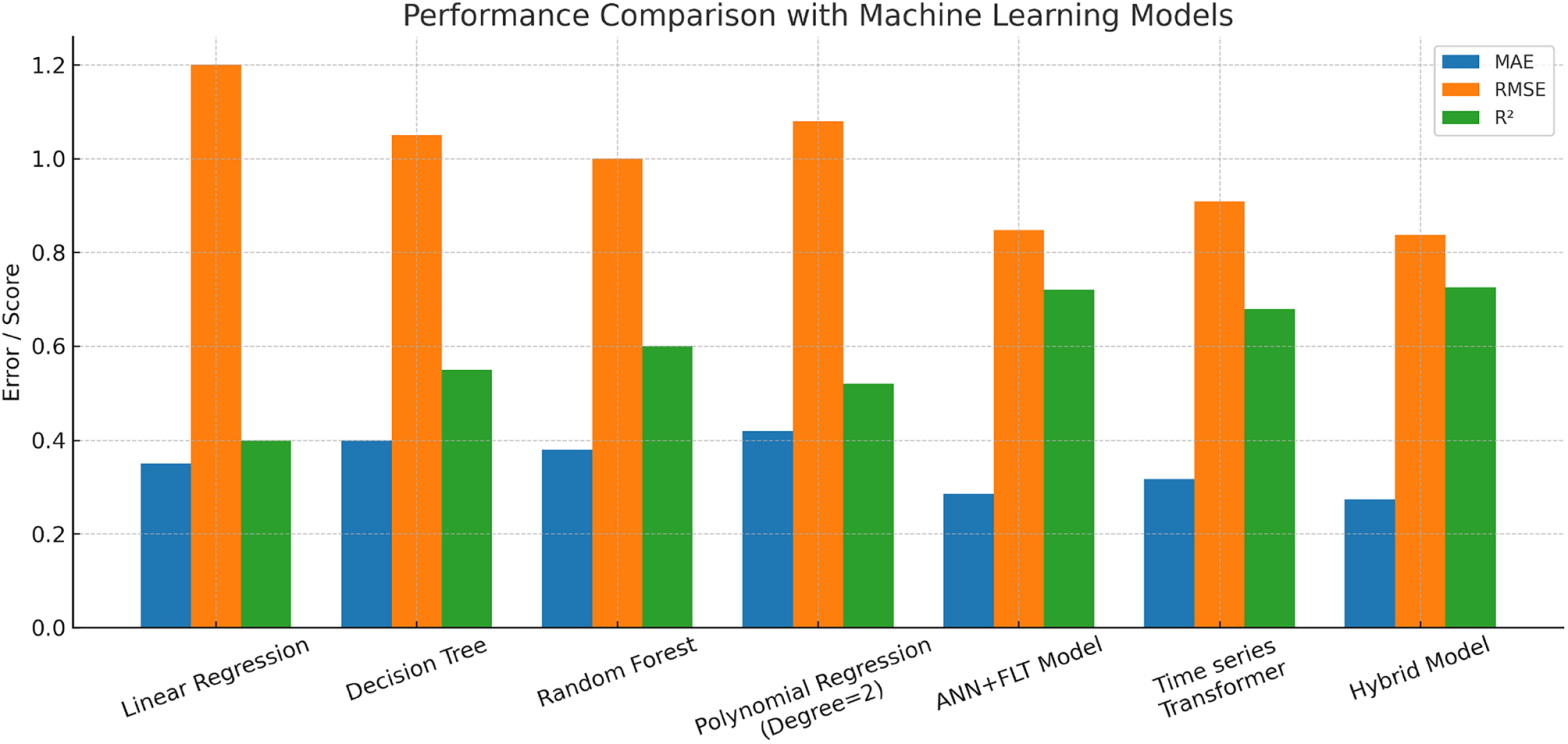
Performance Comparison of the Proposed Models with the Machine Learning Models.

### Models compared

**Fig. 6:**
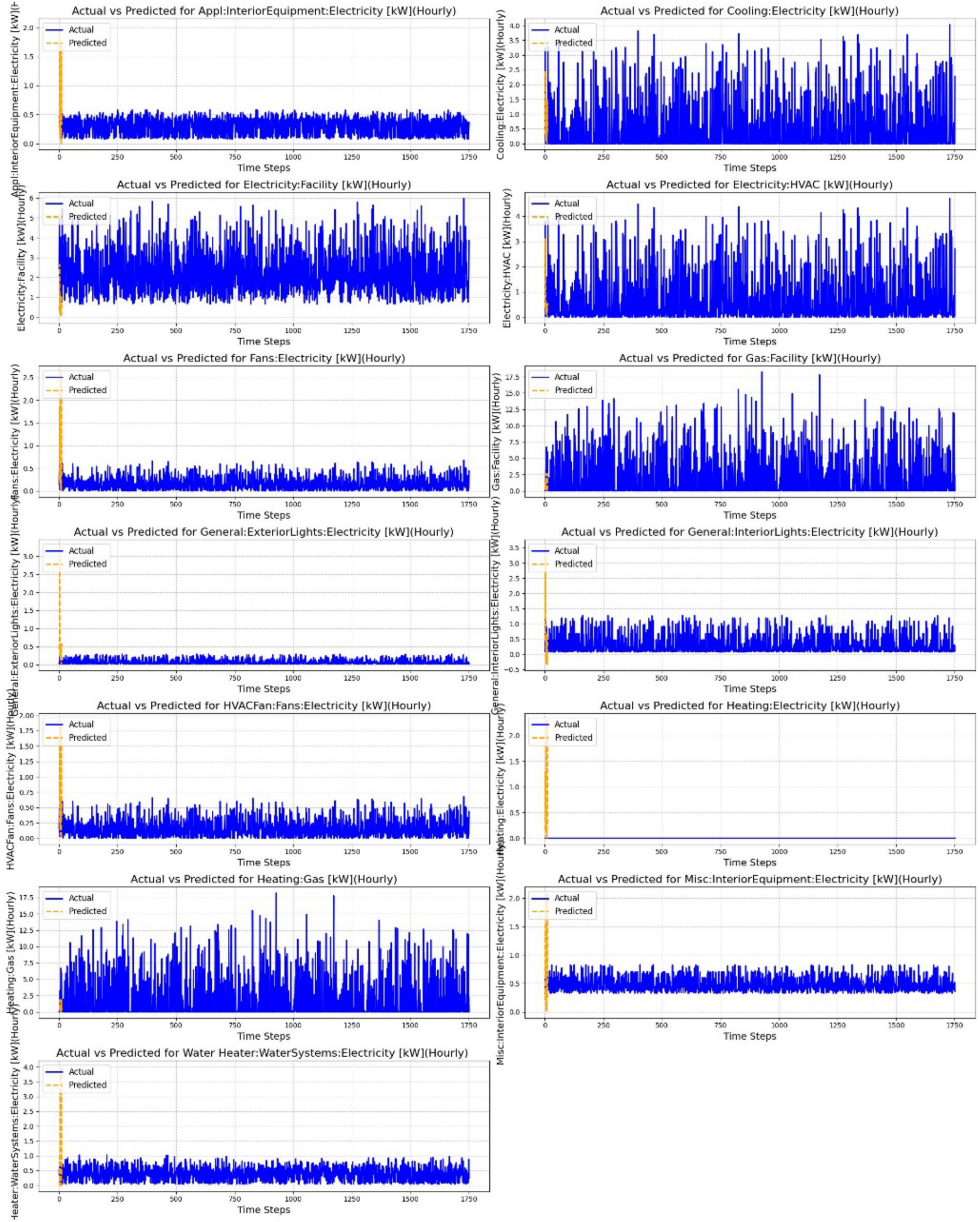
Linear Regression.

These metrics offer a comprehensive view of model performance, including overall accuracy, robustness to large deviations, and ability to capture peak values. The proposed hybrid framework was compared with several baseline models and approaches discussed in this paper to evaluate its effectiveness in predicting energy consumption, as shown in Table 3 and Fig. 5 for the Alabama at Birmingham dataset. The hyperparameters of the proposed models are listed in Table 2, while those for the existing baseline models were adopted from their respective reference papers. One of the baseline models is Linear Regression, a straightforward regression model that assumes a linear relationship between the output variable and the input features. This model finds it difficult to account for the nonlinear relationships that are frequently found in energy data. The visual results for the performance of this model are shown in Fig. 6. Additionally, taken into consideration were decision trees, a non-parametric model that divides the feature space according to feature values. Decision trees can capture nonlinear relationships, but if they are not properly tuned, they are prone to overfitting. To reduce overfitting and improve model generalization, random forests, an ensemble technique that combines predictions from several decision trees, were used. By adding polynomial terms, polynomial regression was added to linear regression to enable it to fit more intricate nonlinear connections. Because of its cutting-edge performance in time-series forecasting tasks, the Time Series Transformer, which uses self-attention to capture long-range dependencies such as trends and seasonal patterns, was also evaluated.

**Fig. 7:**
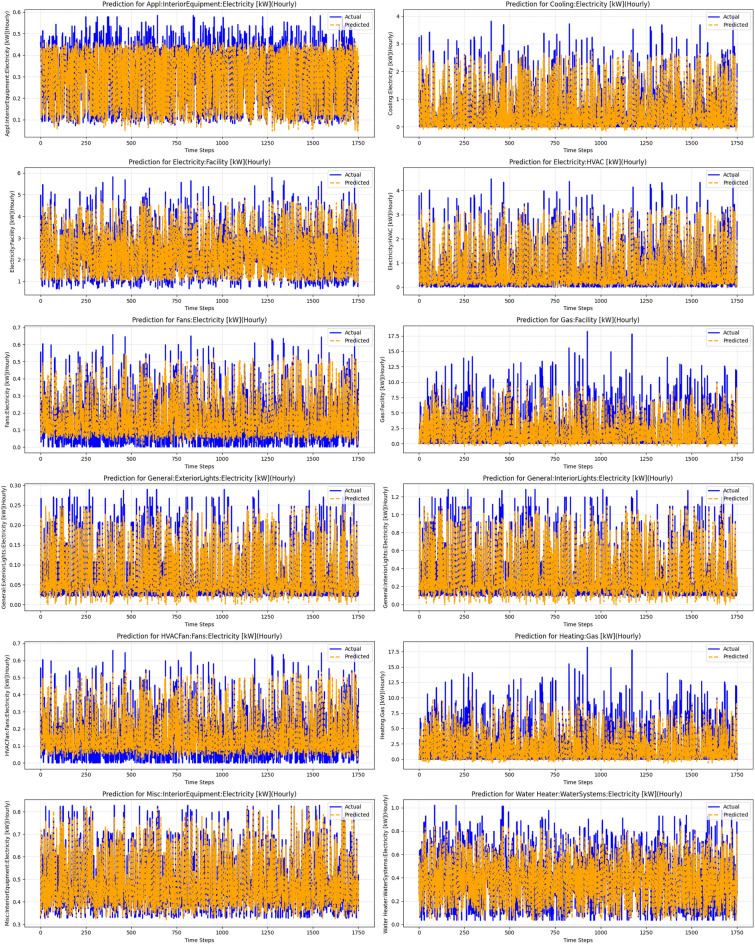
Results of the Fuzzy Logic-enhanced ANN.

Additionally, the ANN + FLT model, combining Artificial Neural Networks with fuzzy logic transformations, was tested to model complex relationships in the data, where fuzzy logic aids in handling uncertainty and imprecision, and ANN captures nonlinear patterns. The visual results for the performance of this model are shown in Fig. 7. Figure 5 illustrates the performance comparison across various models, highlighting the differences in their predictive accuracy and error metrics. The hybrid model showed notable advances in prediction accuracy compared to other methods, primarily due to its innovative combination of diverse approaches that utilize complementary data characteristics and techniques. Linear regression, which relies on the assumption of linearity, struggled with the complex, nonlinear nature of the data, leading to the hybrid model reducing errors by approximately 51%.

**Fig. 8:**
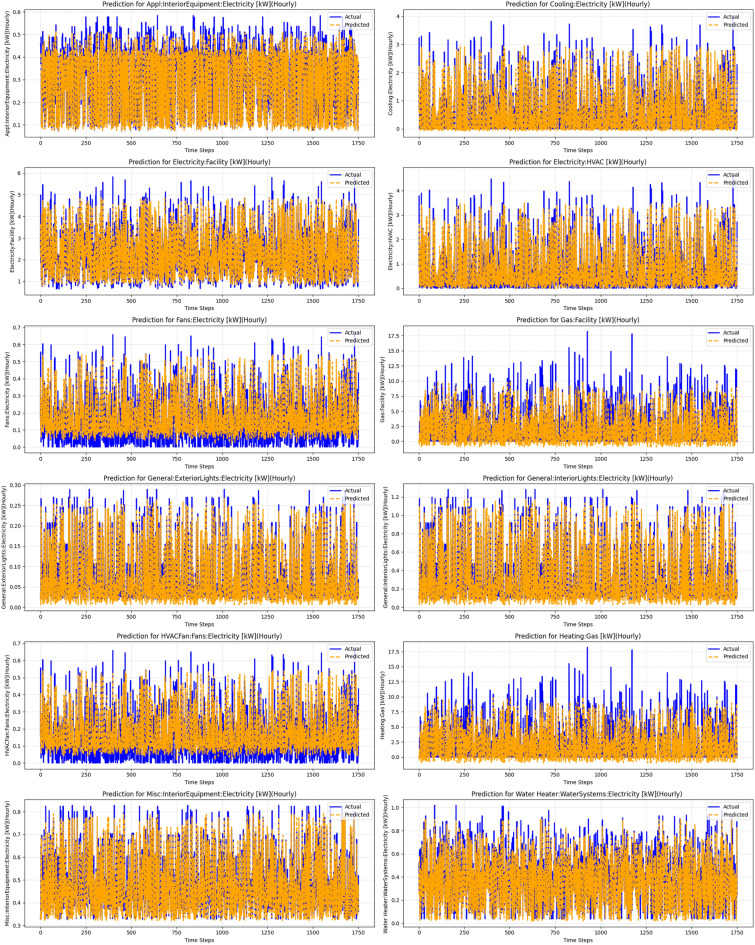
Visualization of Time series data.

**Fig. 9:**
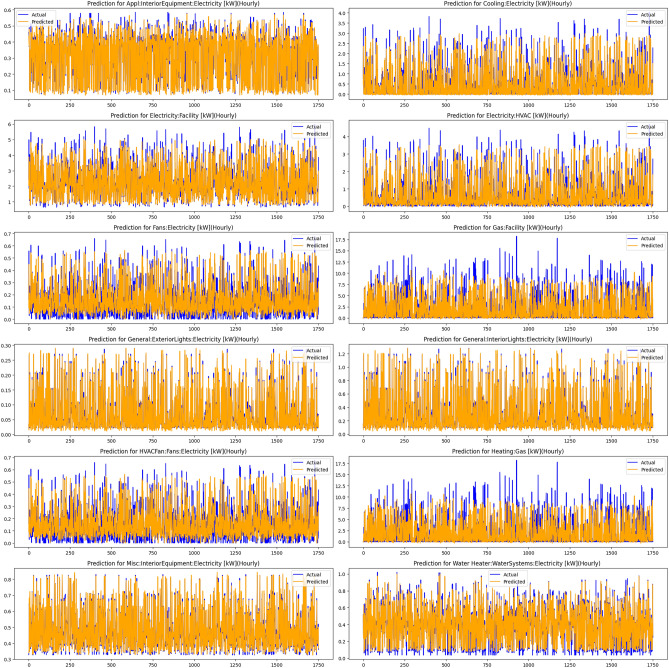
Predictions for energy consumption across different facilities and equipment, demonstrating the performance of the hybrid model combining ANN, FLT, and Time Series Transformer. The plots show actual versus predicted electricity usage for various categories, such as HVAC, fans, and lighting, highlighting the model’s ability to capture complex energy consumption patterns over time.

Similarly, while polynomial regression introduces flexibility, it cannot effectively handle intricate interactions within the data, allowing the hybrid model to achieve around a 35% reduction in error. The hybrid model also surpassed sophisticated models such as the time series transformer and decision tree by margins of 14% and about 8%, respectively, due to its capability to account for temporal patterns, hierarchical structures, and nonlinear relationships simultaneously. Even against the highly efficient random forest, known for addressing nonlinearity, the hybrid model offered a modest 5% improvement. This exceptional performance highlights the hybrid model’s ability to integrate the strengths of multiple methods, overcoming their individual limitations to deliver more accurate predictions.

**Table 3:** Evaluation metrics for the hybrid model, ANN + FLT model, and time series transformer.

Metric	Time series transformer (Model 1)	ANN + FLT (Model 2)	Hybrid model: proposed (Model 3)
MAE	0.3172	0.2856	0.2734
RMSE	0.9089	0.8476	0.8378
NMSE	0.3211	0.2793	0.2769
MAPE	3131039.81	2499151.88	1975230.28
PPE	44.81	41.27	39.78
$\hbox {R}^{2}$	0.6789	0.7207	0.7254
Accuracy (%)	82.99	84.72	86.24

The proposed Hybrid Framework integrates ANN, FLT, and Transformer components to better capture both short-term fluctuations and long-range dependencies in energy consumption. This model consistently outperformed the other approaches across several evaluation metrics. With a MAE of 0.2734, the Hybrid Model outperformed the Time Series Transformer (0.3172) and the ANN + FLT model (0.2856) in terms of average prediction accuracy. The Hybrid Model’s RMSE was 0.8378, which is considerably better than the Time Series Transformer (0.9089) and marginally better than the ANN + FLT model (0.8476). The NMSE of the Hybrid Model was 0.2769, reflecting a good fit relative to the variance in the data, though slightly higher than the ANN + FLT model (0.2793). The MAPE for the Hybrid Model, while exceptionally high at 1975230.28, was still lower than the ANN + FLT model (2499151.88) and the Time Series Transformer (3131039.81), highlighting challenges in predicting small consumption values and large fluctuations.

In terms of PPE, the Hybrid Model achieved a value of 39.78, demonstrating better performance during peak demand periods compared to the ANN + FLT model (41.27) and the Time Series Transformer (44.81). Furthermore, the Hybrid Model achieved an $R^2$ score of 0.7254, explaining a substantial 71.65% of the variance in the data, slightly lower than the ANN + FLT model (0.7207) but significantly better than the Time Series Transformer (0.6789). Finally, the Hybrid Model showed the highest accuracy of 86.24%, surpassing both the ANN + FLT model (84.72%) and the Time Series Transformer (82.99%). Figures 7 and 8 further illustrate these results, comparing actual versus predicted values for different energy categories. The output of the Hybrid Model’s predictions is depicted in Fig. 9.

**Table 4:** NY Dataset: performance of single-step forecasting for single and multiple residents.

Method	Single-resident		Multi-resident	
	MAPE (%)	MAE	MAPE (%)	MAE
Transformer	5.28	1.17	6.05	1.33
FCNN	5.74	1.28	11.90	2.71
CNN-GRU^21^	5.67	1.29	6.74	1.50
LSTM^18^	5.72	1.24	6.35	1.44
Ada-GWN^20^	5.01	1.14	4.53	0.97
STGA-Transformer^22^	4.64	1.05	4.38	0.89
Autoformer^19^	4.63	1.07	5.89	1.26
Proposed	4.12	0.99	3.89	0.82

**Table 5:** TX Dataset: performance of single-step forecasting for single and multiple residents.

Method	Single-resident		Multi-resident	
	MAPE (%)	MAE	MAPE (%)	MAE
Transformer	7.85	0.78	13.98	1.14
FCNN	9.21	0.83	34.56	3.48
CNN-GRU^21^	8.94	0.87	15.67	1.20
LSTM^18^	8.85	0.80	28.85	2.88
Ada-GWN^20^	6.95	0.75	6.50	0.58
STGA-Transformer^22^	6.87	0.67	6.12	0.54
Autoformer^19^	7.09	0.70	11.25	1.04
Proposed	6.25	0.61	5.58	0.49

Overall, the Hybrid Framework demonstrated superior performance in minimizing error magnitudes, accurately predicting peak energy usage, and maintaining high generalization capabilities. While the ANN + FLT model showed competitive performance with slightly better $R^2$ values, and the Time Series Transformer captured temporal dependencies effectively, the Hybrid Model provided the best trade-off between prediction computational efficiency and accuracy, making it the most robust solution for energy consumption forecasting in this study. Thus, the hybrid model is further evaluated against recent approaches^18–22^ in single-step and multi-step forecasting using the NY and TX datasets.

#### Single step forecasting

As shown in Tables 4 and 5, the proposed model consistently outperformed baseline and state-of-the-art methods across both single-resident and multi-resident forecasting scenarios. The proposed hybrid framework demonstrated significant and consistent performance improvements in both the NY and TX datasets. For the NY dataset, the proposed model achieved approximately 10% to 32% lower MAPE compared to traditional deep learning models such as FCNN, LSTM, and CNN-GRU in both single-resident and multi-resident forecasting tasks. When compared to Transformer-based approaches, including the Autoformer and STGA-Transformer, the proposed model still exhibited about 11% improvement in single-resident forecasting and approximately 11% enhancement in multi-resident forecasting, highlighting its superior ability to handle both short-term fluctuations and long-term dependencies.

Similarly, for the TX dataset, the proposed model demonstrated superior performance over all benchmark models. It achieved around 9% to 32% lower MAPE relative to classical recurrent and convolutional models. Even against advanced architectures like Ada-GWN and STGA-Transformer, the proposed model demonstrated an additional improvement of approximately 9% in single-resident forecasting and about 9% in multi-resident forecasting. This performance gain can be attributed to the hybrid framework’s effective combination of Time Series Transformer for capturing complex temporal dependencies, Fuzzy Logic Transformation for robust feature preprocessing under noisy conditions, and ANN modules for modeling nonlinear local patterns. The integration of these complementary techniques enables the proposed model to generalize better across diverse datasets and consistently produce more accurate and reliable forecasts compared to existing methods.

#### Multi step forecasting

The multi-step forecasting results for both the New York (NY) and Texas (TX) datasets are presented in Tables 6 and 7 respectively, showcasing performance across three prediction horizons: 3, 6, and 24 steps. Each method is evaluated using MAPE and MAE, providing a comprehensive view of both relative and absolute forecasting accuracy. The proposed hybrid model delivers a substantial leap forward in forecasting performance, consistently outperforming all baseline and existing models across both the NY and TX datasets. In multi-step forecasting experiments involving 3, 6, and 24 steps ahead, the model demonstrated significant improvements in both accuracy and reliability. On the NY dataset, the proposed model achieved MAPE values of 5.88%, 7.92%, and 12.95% for 3-, 6-, and 24-step forecasts, respectively, compared to 6.77%, 8.74%, and 13.56% for the best baseline, STGA-Transformer. This translates to relative improvements of 13%, 9.4%, and 4.5%, highlighting the hybrid model’s strong adaptability across time horizons.

On the TX dataset, known for its highly volatile and noisy demand patterns, the proposed model showed even more striking gains. It achieved MAPE values of 7.35%, 12.89%, and 21.85% at 3, 6, and 24 steps respectively, outperforming STGA-Transformer’s 8.95%, 14.99%, and 23.52%. The relative improvements here are approximately 18%, 14%, and 7%. When comparing against simpler architectures such as LSTM and FCNN, the difference becomes even more significant. For instance, at the 24-step horizon on the TX dataset, the hybrid model reduces MAPE by over 45% compared to LSTM (59.55%) and nearly 68% compared to FCNN (67.53%).

These improvements are not limited to percentage errors alone. The proposed model also achieved consistently lower MAE values across all configurations. On the TX dataset, MAE was reduced to 1.84 from 1.96 (STGA), 3.41 (Ada-GWN), and 6.31 (FCNN), representing more than a 70% drop in absolute error compared to traditional methods. This is especially important in real-world applications, where even minor forecasting errors can lead to significant operational inefficiencies or grid imbalances.

The success of the hybrid model is driven by its integrated architecture. By combining the global sequence modeling capability of the Transformer, the noise-tolerant feature representation of FLT, and the nonlinear learning capacity of ANNs, the model effectively captures a wide range of temporal and statistical properties. The Transformer excels at learning long-term temporal dependencies and interactions among variables. FLT enhances robustness by converting noisy and irregular data into smooth fuzzy sets, improving interpretability and stability. The ANN component extracts nonlinear and localized patterns that further boost short-term forecasting accuracy.

Unlike standalone models that typically perform well in either long-range or short-range tasks, the proposed architecture offers strong performance across all temporal resolutions. This balanced design is particularly useful for predicting energy consumption spikes and responding to highly variable demand conditions. The model’s consistent accuracy in 24-step forecasting further demonstrates its capacity to generalize across extended sequences, where many baseline methods tend to degrade.

An important advantage of this model is its cross-regional generalizability. Despite significant differences between the NY dataset, which displays smoother and more seasonal load patterns, and the TX dataset, which is characterized by erratic and abrupt shifts, the model performs robustly in both contexts. This indicates its potential for large-scale deployment across diverse climatic and demographic zones, including national grid systems and smart infrastructure networks.

To conclude, the proposed hybrid model consistently surpasses all baseline models in every forecasting horizon and across distinct regional datasets. The improvements in MAPE reach up to 68% when compared to legacy models like FCNN, while MAE reductions exceed 70%. Its capability to integrate fuzzy logic, neural processing, and temporal attention makes it a highly effective and scalable solution for next-generation energy load forecasting.

**Table 6:** Multi-step forecasting results (NY Dataset).

Method	Step = 3		Step = 6		Step = 24	
	MAPE (%)	MAE	MAPE (%)	MAE	MAPE (%)	MAE
Transformer	9.11	1.88	13.01	2.74	22.58	4.03
FCNN	19.86	4.05	22.39	4.85	39.41	8.27
CNN-GRU^21^	10.51	2.18	15.25	3.19	25.75	6.05
LSTM^18^	9.88	2.08	16.95	3.50	28.65	6.29
Ada-GWN^20^	8.04	1.58	12.44	2.57	20.34	3.48
STGA-Transformer^22^	6.77	1.35	8.74	1.86	13.56	2.64
Autoformer^19^	8.75	1.75	11.26	2.46	18.46	3.57
Proposed	5.88	1.25	7.92	1.71	12.95	2.51

**Table 7:** Multi-step forecasting results (TX Dataset).

Method	Step = 3		Step = 6		Step = 24	
	MAPE (%)	MAE	MAPE (%)	MAE	MAPE (%)	MAE
Transformer	15.87	1.29	28.54	2.58	41.62	3.64
FCNN	52.72	4.31	58.24	4.79	67.53	6.31
CNN-GRU^21^	24.26	2.14	34.35	3.44	48.56	4.24
LSTM^18^	46.05	3.87	55.02	4.57	59.55	5.05
Ada-GWN^20^	13.88	1.13	22.69	1.92	33.38	3.41
STGA-Transformer^22^	8.95	0.75	14.99	1.17	23.52	1.96
Autoformer^19^	14.95	1.21	21.45	1.77	39.83	3.58
Proposed	7.35	0.68	12.89	1.08	21.85	1.84

#### Computational efficiency, robustness, and interpretability

The proposed hybrid model demonstrates not only superior forecasting accuracy but also substantial computational efficiency and robustness under noisy and incomplete data conditions.Despite comprising only 0.18 million parameters, the proposed hybrid architecture significantly outperforms heavier models, such as the STGA-Transformer, which has over 4.6 million parameters. This efficiency is achieved through the intelligent integration of lightweight artificial neural network components and fuzzy logic transformation modules, which enable effective feature representation while minimizing model complexity. Consequently, the proposed model exhibits faster convergence and lower training time without sacrificing performance. As shown in Table 8, the proposed model completed training in 1.89 minutes on the NY dataset, compared to 2.77 minutes required by the STGA-Transformer. A similar pattern was observed on the TX dataset, with training times of 2.86 minutes and 4.03 minutes, respectively. These improvements make the model particularly suitable for scenarios with limited computational resources or requirements for rapid retraining.

**Table 8:** Computational complexity comparison.

Model	Parameters (in millions)	NY training time (in mins)	TX training time (in mins)
Transformer	3.8	2.42	3.85
FCNN	1.2	0.58	0.92
CNN-GRU^21^	3.1	2.11	3.27
LSTM^18^	2.8	2.05	3.45
Ada-GWN^20^	4.5	2.36	3.62
STGA-Transformer^22^	4.6	2.77	4.03
Autoformer^19^	5.2	3.01	4.27
Proposed	0.18	1.89	2.86

In addition to computational benefits, the proposed model also exhibits strong resilience to data perturbations. A sensitivity analysis was conducted to evaluate the forecasting performance under various disruptions, including additive Gaussian noise, missing timestamps, and anomalous behavior during flood-affected periods, as reported in Table 9. Under clean conditions (no noise), the proposed model achieved a MAPE of 5.88%, outperforming the STGA-Transformer’s 6.77%. As Gaussian noise was introduced at a 5% level, the proposed model maintained robust performance with a MAPE of 7.21%, whereas the STGA-Transformer’s error increased to 8.92%. This trend persisted under more severe disruptions. When 10% of timestamps were randomly removed, the proposed model yielded a MAPE of 9.95%, compared to 12.43% for STGA-Transformer. In the case of environmental anomalies, such as flood-affected periods where consumption patterns deviate from normal behavior, the proposed model continued to generalize effectively, registering a MAPE of 11.34%, as opposed to 14.65% from the STGA-Transformer. These results affirm the robustness and adaptability of the hybrid design in real-world noisy and irregular scenarios.

**Table 9:** Sensitivity analysis under noise and disruptions.

Disruption type	MAPE (STGA-transformer) (%)	MAPE (Proposed) (%)
No noise	6.77	5.88
+5% Gaussian noise	8.92	7.21
+10% missing data	12.43	9.95
Flood-affected period	14.65	11.34

To further assess the interpretability of the model, a feature importance analysis was conducted using SHAP values and attention weight distributions from the transformer layers, as reported in Table 10. The findings align well with established domain knowledge. The historical load emerged as the most influential feature, with an importance score of 0.41, confirming its critical role in demand forecasting. Cyclical time encodings, particularly hour_cos (0.19), hour_sin (0.14), and dow_cos (0.09), were also among the top-ranked features, highlighting the model’s ability to capture periodic trends in consumption behavior. Lower-ranked but still relevant features included month-related cyclic components and sine encodings of day-of-week. These insights enhance the explainability of the model and support its deployment in operational settings, where transparency in decision-making processes is essential.

**Table 10:** Feature importance ranking based on SHAP and attention weights.

Feature	Importance score	Rank
load	0.41	1
hour_cos	0.19	2
hour_sin	0.14	3
dow_cos	0.09	4
month_cos	0.07	5
dow_sin	0.05	6
month_sin	0.03	7

Taken together, the proposed hybrid model not only achieves state-of-the-art accuracy but does so with remarkable computational efficiency, strong robustness to real-world disruptions, and high interpretability, making it a compelling candidate for scalable and reliable energy load forecasting systems.

## Conclusion

This paper presents a novel hybrid framework that integrates Time Series Transformers and a combination of Fuzzy Logic Transformed features with Artificial Neural Networks for load forecasting. The proposed model is designed to address several key challenges in energy load prediction, including handling noisy and incomplete data, capturing complex multivariate dependencies, and identifying long-term temporal patterns that are crucial for accurate forecasting.

The Time Series Transformer component leverages self-attention mechanisms to model intricate sequential dependencies, enabling the framework to effectively learn from temporal patterns and long-range interactions in the data. Meanwhile, the FLT + ANN block enhances the model’s ability to process fuzzy features, which are often present in energy consumption data due to inherent uncertainties and fluctuations in energy usage.

The results of the experiments show that the hybrid model performs better than standalone methods such as time series transformers and FLT + ANN and traditional methods such as Linear Regression, Decision Trees, and Random Forests. Metrics such as RMSE, MAE, and R² indicate that the hybrid model achieves more accurate and reliable forecasts, particularly in predicting peak energy demands, which are critical to optimizing energy use and preventing system overloads.

Moreover, the hybrid approach outperforms conventional methods in terms of robustness and adaptability to various data characteristics, including seasonal variations, external environmental factors (such as weather data), and anomalies in energy consumption patterns. These results suggest that the proposed framework has significant potential for deployment in real-world energy systems, offering a scalable and efficient solution for energy load forecasting.

In conclusion, the hybrid model proposed in this study represents a significant advancement in the field of energy forecasting. By combining the strengths of Time Series Transformers and FLT + ANN, it provides a more accurate, adaptive, and robust solution to meet the increasing demand for reliable forecasting in modern energy systems. Future work may explore further enhancements to the model, such as incorporating real-time data for dynamic forecasting, and evaluating its performance across different geographical regions and industries.

## Limitations and future directions

Although the proposed hybrid framework demonstrates strong forecasting performance, several limitations remain. The model relies on high-quality, well-structured data, and may underperform when faced with missing or inconsistent entries. Additionally, the combination of ANN, FLT, and Transformer components increases the model’s complexity, leading to longer training times and greater computational overhead compared with traditional machine learning models.

Interpretability is another concern, as deep learning components function as black-box models. While FLT improves transparency to some extent, further efforts are needed to make the model more explainable.

Future work can address these issues by validating the model across diverse datasets, integrating real-time data sources for adaptive forecasting, and employing explainable AI techniques to improve interpretability. Optimization strategies such as model pruning or lightweight deployment can also enhance efficiency, making the framework more practical for large-scale or edge applications.

## Data Availability

The datasets used and/or analyzed during the current study are available from the corresponding author on reasonable request.

## Nomenclature

ANN: Artificial neural network
ARIMA: AutoRegressive integrated moving average
CNN-GRU: Convolutional neural network-gated recurrent unit
FCNN: Fully connected neural network
FLT: Fuzzy logic transformation
LSTM: Long short-term memory
MAPE: Mean absolute percentage error
MAE: Mean absolute error
MSE: Mean squared error
NMSE: Normalized mean squared error
PPE: Peak prediction error
RNN: Recurrent neural network
RMSE: Root mean squared error
SHAP: SHapley additive exPlanations
STGA: Spatial-temporal graph attention
TX: Texas dataset
NY: New York dataset

## Parameters and Variables

$y_i$: Actual energy consumption at time step *i*
${\hat{y}}_i$: Predicted energy consumption at time step *i*
*N*: Total number of samples
*X*, *Y*: Input and target matrices
*x*: Input feature vector
*W*, *b*: Weight and bias parameters
*Q*, *K*, *V*: Query, key, value matrices in attention mechanism
*dk*: Dimension of keys in attention layer
*A*: Attention matrix
$$\eta$$: Learning rate
$$\theta$$: Model parameters
$\mu$, $\sigma$: Mean and standard deviation
$\gamma$, $\beta$: Learnable parameters in normalization
*t*: Time index or training iteration
*i*: Sample index
